# Low-Educated Women with Chronic Pain Were Less Often Selected to Multidisciplinary Rehabilitation Programs

**DOI:** 10.1371/journal.pone.0097134

**Published:** 2014-05-21

**Authors:** Anne Hammarström, Inger Haukenes, Anncristine Fjellman Wiklund, Arja Lehti, Maria Wiklund, Birgitta Evengård, Britt-Marie Stålnacke

**Affiliations:** 1 Department of Public Health and Clinical Medicine, Umeå University, Umeå, Sweden; 2 Umeå Centre for Gender Studies in Medicine, Umeå University, Umeå, Sweden; 3 Division of Mental Health, Department of Public Mental Health, Norwegian Institute of Public Health, Bergen, Norway; 4 Department of Community Medicine and Rehabilitation, Physiotherapy, Umeå University, Umeå, Sweden; 5 Department of Clinical Microbiology, Division of Infectious Diseases, Umeå University, Umeå, Sweden; 6 Department of Community Medicine and Rehabilitation, Rehabilitation Medicine, Umeå University, Umeå, Sweden; University of Washington, United States of America

## Abstract

**Background:**

There is a lack of research about a potential education-related bias in assessment of patients with chronic pain. The aim of this study was to analyze whether low-educated men and women with chronic pain were less often selected to multidisciplinary rehabilitation than those with high education.

**Methods:**

The population consisted of consecutive patients (n = 595 women, 266 men) referred during a three-year period from mainly primary health care centers for a multidisciplinary team assessment at a pain rehabilitation clinic at a university hospital in Northern Sweden. Patient data were collected from the Swedish Quality Registry for Pain Rehabilitation National Pain Register. The outcome variable was being selected by the multidisciplinary team assessment to a multidisciplinary rehabilitation program. The independent variables were: sex, age, born outside Sweden, education, pain severity as well as the hospital, anxiety and depression scale (HADS).

**Results:**

Low-educated women were less often selected to multidisciplinary rehabilitation programs than high-educated women (OR 0.55, CI 0.30–0.98), even after control for age, being born outside Sweden, pain intensity and HADS. No significant findings were found when comparing the results between high- and low-educated men.

**Conclusion:**

Our findings can be interpreted as possible discrimination against low-educated women with chronic pain in hospital referrals to pain rehabilitation. There is a need for more gender-theoretical research emphasizing the importance of taking several power dimensions into account when analyzing possible bias in health care.

## Introduction

A large number of studies indicate that there is a gender bias to women's disadvantage, i.e. an unintended and systematic neglect of women, in health care [1], [2]. Most of this research has been performed on coronary heart disease [3], but also in relation to other symptoms and diagnoses [1], [4]–[6]. For example, the so-called “laundry bag project” (LBP) discovered gendered standards for dermatological treatment of common diagnoses. The study included gender-based quantitative analysis of treatment of all patients (n = 320 women, 421 men) referred to a dermatological clinic. The study showed that men with diagnoses of psoriasis or eczema received more whole-body UV treatment and more help with emollients than did women [5]. In economic terms, women patients subsidized the treatment budget of the clinic to a value of 22 per cent. In similar ways, medically unjustified differences in the availability of examination and treatment for women compared to men have been demonstrated in connection with a number of other diseases, such as irritable bowel syndrome [1], renal transplantation, HIV and pain [6], [7]. Gender bias in neck pain was found when Swedish interns were asked about the diagnosis and management of this group of patients. Non-specific somatic diagnoses, psychosocial questions, drug prescriptions and the expressed need of diagnostic support from a physiotherapist and an orthopedist were more commonly proposed for women than for men [7].

Gender bias may also mean that men are disadvantaged in health care [8], which has been discussed for example in relation to the treatment of depression [9] and osteoporosis [10] in older men. In these cases, diagnostic models have been developed for women while criteria to identify risk in men are not well established. A case study of osteoporosis from Gendered Innovations [10] has developed male reference populations and identified medical conditions (especially among men) that are related to osteoporotic fracture, allowing for better evaluation of fracture risk in men. In addition, among patients with chronic pain, women participate in multidisciplinary rehabilitation programs (which are a combination of different physical and psychological interventions that is linked to teamwork) more often than men, and some studies have demonstrated that women benefit more from this kind of rehabilitation than men do [11]. Systematic reviews of treatment and rehabilitation of patients with chronic pain have shown evidence that multidisciplinary rehabilitation programs have superior effects on multidimensional outcomes compared to less intensive treatments [12]–[14]. However, most of the reviews do not analyze differences in treatment among men as compared to women. Thus, the question remains to study whether there is a gender bias in the treatment of chronic pain.

Increasingly important has been to analyze not only gender, but to include multiple power dimensions in the analyzes of gender bias such as socioeconomic status, ethnicity and age. For example in Swedish health care, research on gender bias has shown that it is not women as a group but older, low-educated women who have worse outcome in stroke care [15]. However, overall rather few studies have been performed within this field of intersectional gender research. To the best of our knowledge there is no research analyzing whether or not low-educated women (and men) of various ages with chronic pain are less frequently selected for multidisciplinary rehabilitation compared to high-educated.

The aim of this study was therefore to analyze whether low-educated men and women with chronic pain were less often selected for multidisciplinary rehabilitation than those with high education.

## Methods

### Ethical statement

The study was approved by the Regional Ethics Vetting Board in Umeå, Sweden. Informed consent was not required because we only handled unidentified register data. According to Swedish law (Swedish Ethical Review Act 2003;460, §§ 20–21, Swedish Personal Data Law 1998:204 § 19) informed consent is not required when dealing with unidentified register data (as was the case in our study).

### Setting

The study was conducted in a clinical setting at the Pain Rehabilitation Clinic at Umeå University Hospital, Sweden. In order for a referred patient to be selected for assessment at the clinic the patients had to have a chronic disabling non-malignant diagnosis of chronic pain. Patients with serious somatic diagnoses (such as cancer, rheumatoid arthritis and neurological disorders that should be investigated by other specialist clinics) are excluded. The most frequent diagnostic groups at the clinic are columnar pain (50%) followed by extremity pain (18%) [16].

The selected patients were assessed during two days at the pain rehabilitation clinic by multidisciplinary diagnostic teams consisting of a specialist physician in rehabilitation medicine, a physiotherapist, a social worker, an occupational therapist and a psychologist if needed. If the multidisciplinary teams assessed that the patient was in need of multidisciplinary rehabilitation and fulfilled the inclusion criteria (described below), they were selected to participate in a rehabilitation program based on a bio-psychosocial model with cognitive behavioural principles [16]. The multidisciplinary rehabilitation program focused on pain management and education about pain and its consequences. Rehabilitation was based on collaboration within the multidisciplinary team with the patient as an active team member. The patient was expected to participate with the team in goal setting and reaching the decided goals. A number of core sessions were conducted, e.g. physiotherapy (swimming pool exercise and relaxation exercises), ergonomics, education about pain mechanisms and coping with pain. At the end of the program contact was established with the patient's primary care physician.

Inclusion criteria for referral to the multidisciplinary rehabilitation program were (*i*) disabling non-malignant chronic and complex musculoskeletal pain (on sick leave or experiencing major interference in daily life due to chronic pain); (*ii*) age 18–65 years; (*iii*) no further medical investigations needed; (*iv*) written consent to participate in and attend the multidisciplinary program; (*v*) agreement not to have parallel contacts with therapists such as physiotherapists while attending the multidisciplinary pain rehabilitation program.

### Population

The population consisted of consecutive patients (n = 595 women, 266 men) referred mainly from primary health care centers to the pain rehabilitation clinic and assessed between 5 November 2007 and 13 December 2010.

### Design and data collection

Patient data were collected from the Swedish Quality Registry for Pain Rehabilitation National Pain Register (SQRP) [17] and linked to the patients' individual records containing the final decision on being selected or not to multidisciplinary rehabilitation programs. The SQRP register has aggregated data since 1998 of all patients referred to the majority of Swedish rehabilitation units. The SQRP is based on patients' information from validated self-administered questionnaires completed before the first multidisciplinary assessment [17]. The patients completed the set of questionnaires at home the night before the assessment and the questions refer to pain experiences during the day before the assessment. The questionnaires were handed in on the day of assessment and were subsequently registered in SQRP.

### Outcome variable

The outcome variable was being selected ( = 1) for a multidisciplinary rehabilitation program as compared to not being selected ( = 0).

### Independent variables

The following independent variables were used: sex, age (used as a continuous variable) and country of birth (Sweden, other Nordic country, Europe (except Nordic countries) and other country) recoded as born outside of Sweden = 1, born in Sweden = 0. Education was measured with the following question: Which is your highest completed level of education? The following four answer alternatives were given: 1. Nine years of compulsory school, 2. Two- or three-year secondary high school (including both theoretical programs and vocational training), 3. University studies 4. Other education (which could mean in-service training supported by a company or organization, folk high school etc.). Low-educated was defined as having completed compulsory school ( = 1) as compared to all other completed forms of education.

To adjust for depression and anxiety, the often used and validated 14-item self-reported HADS (Hospital, anxiety and depression) scale was used [18]. Due to high correlation between the anxiety and the depression scale, the two scales were combined into a continuous variable with a total range of 0–42 [19]. In the multivariate logistic regression analyzes, low HADS equals 0.The HADS has proven to be reliable and valid when used to assess symptom severity in anxiety and depression among somatic, psychiatric and primary care patients [20].

Pain severity was used as a continuous variable (range 0–6) based on a subscale from the Multidimensional Pain Inventory (MPI), Part I [21], [22]. In the multivariate logistic regression analyzes, low pain severity equals 0. The MPI has demonstrated good reliability and validity for patients with chronic pain [23].

### Statistical analysis

The associations between low education and referral to multidisciplinary rehabilitation programs were investigated for men and women separately by means of multivariate logistic regression analyzes, using SPSS statistical package (SPSS version 18 for Windows). The first model (Model 0) consisted of bivariate associations. The following models were age-adjusted. Model 1 included the variable ‘born outside Sweden’ while model 2 also included HADS and pain severity. As significance tests we used chi-square for dichotomous variables and t-test for continuous variables. The correlation between the confounders was <0.3.

### Availability of data

The SQRP is a national quality registry supported by the Swedish Association of the Local Authorities and Regions and connected to the Uppsala Clinical Research Center (UCR). Our dataset has great potential for secondary analysis. The data are not freely available but collaborative ideas are welcome. Britt-Marie Stålnacke is the contact person. The website with documentation for the SQRP and detailed information about variables is available at http://www.ucr.uu/nrs/.

## Results

The distribution of the dependent and independent variables for men and women are shown in Tables 1 and 2.

**Table 1:** Prevalence of dichotomous covariates among women and men, n = 861 (per cent).

	Women n = 595	n	Men n = 266	n	p-value
Multidisciplinary rehabilitation	28.4	169	18.4	49	0.002
Low-educated	16.1	96	14.4	38	0.542
Born outside Sweden	9.4	56	14.7	39	0.026

**Table 2:** Prevalence of continuous covariates between women and men, n = 861 (mean (SD)).

	Women n = 595	Men n = 266	p-value
Age	39.9 (10.9)	40.8 (10.9)	0.301
HADS	14.9 (8.1)	15.0 (8.4)	0.810
Pain severity	4.4 (0.9)	4.3 (0.9)	0.402

The tables show that significantly more women than men were selected for multidisciplinary rehabilitation. More men than women were born abroad. For the other variables, no significant differences between men and women were found. Around 15 per cent were low-educated.

Table 3 shows the logistic regression analyzes in four age-adjusted models for men and women separately with referral to multidisciplinary rehabilitation programs as outcome.

**Table 3:** Logistic regression analyzes for referral to multidisciplinary rehabilitation program among women and men in relation to low education (reference = high education) and other independent variables.

	Model 0				Model 1				Model 2			
	Women		Men		Women		Men		Women		Men	
	OR	95% CI	OR	95% CI	OR	95% CI	OR	95% CI	OR	95% CI	OR	95% CI
High-educated	1			1		1		1		1		1
**Low-educated**	**0.53**	**0.31–0.92**	**0.81**	**0.32–2.05**	**0.53**	**0.31–0.93**	**0.81**	**0.32–2.10**	**0.55**	**0.30–0.98**	**0.88**	**0.34–2.30**
Born in Sweden	1			1		1		1		1		1
Born outside Sweden	0.39	0.18–0.85	0.61	0.23–1.65	0.41	0.19–0.89	0.65	0.24–1.76	0.67	0.30–1.51	0.90	0.30–2.69
Low HADS	1			1						1		1
High HADS	0.97	0.95–1.00	1.03	1.00–1.07					0.98	0.96–1.00	1.03	0.99–1.08
Low pain severity	1			1						1		1
High pain severity	0.79	0.64–1.00	0.92	0.65–1.29					0.86	0.69–1.08	0.93	0.64–1.36

Model 0: bivariate associations.

Model 1: adjusted for age + born outside Sweden.

Model 2: adjusted for age, born outside Sweden + HADS and pain severity.

The table shows that low-educated women were less often selected for multidisciplinary rehabilitation programs as compared to high-educated women. The odds ratios for low education were significant in all models and did not particularly attenuate in the fully adjusted model (from 0.53 in the univariate to 0.55 in the last model). Among men, there were no significant odds ratios between low education and referral to multidisciplinary rehabilitation programs in any of the models. But the odds ratios pointed in the same direction as among women. None of the other independent variables were significantly related to multidisciplinary rehabilitation among men or women.

## Discussion

This study aimed to analyze whether low-educated men and women with chronic pain were less often selected for multidisciplinary rehabilitation compared to high-educated. We found that low-educated women were less often selected for multidisciplinary rehabilitation programs than high-educated women and that this relationship remained almost unchanged after control for all the covariates (including pain intensity and mental illness).

A possible explanation to these findings may be that women with lower levels of education might be less likely to fulfil the inclusion criteria. Women might for example be more likely to need further medical investigations or be less likely to agree to give up their contacts with other. However, neither low-educated nor women were overrepresented among those who needed further medical investigation (data not shown). In addition, almost everyone who was selected to the multidisciplinary rehabilitation programs agreed to participate. Thus, the fact that low-educated women were not referred to multidisciplinary rehabilitation as often as high-educated women cannot be explained by such factors.

Overall, there is a lack of international studies about possible bias in referral of low-educated patients to pain rehabilitation. However, our findings are in line with a broader scope of research, demonstrating socioeconomic bias in specialist health care [24]–[28]. A comprehensive study of health care utilization in 12 EU member states found consistent evidence that the wealthy and/or high-educated were more likely to have contact with medical specialists than the poor and low-educated [28]. Moreover, selection for cardiac rehabilitation has been found to favor participants with good prognosis and disfavor patients from deprived areas who tend to have poorer prognosis [24], [26]. Also waiting time for carotid surgery after stroke was significantly longer for low-income patients compared with high-income patients [27], [29]. However, gender differences were not analyzed in these studies.

The current finding that low-educated women with chronic musculoskeletal pain were less often selected for multidisciplinary rehabilitation programs is surprising for several reasons. First, consistent findings point to socioeconomic indicators, such as educational level, as strong predictors of musculoskeletal disorders and reporting of chronic pain conditions in both men and women [30]–[34]. MacFarlaine et al. found that low socioeconomic status in adulthood was associated with major regional musculoskeletal pain and chronic widespread pain [32]. Individuals in the lowest socioeconomic class had a three-fold increased risk of widespread pain, and the impact of childhood socioeconomic status was less prominent than adult socioeconomic status [32]. In addition Overland et al. found that individuals with widespread musculoskeletal pain were characterized by being women, having lower education/lower household income, poor general health including higher prevalence of common mental disorders and higher risk for future disability pension [35]. Based on these findings one should expect that lower educated women and men with chronic musculoskeletal pain conditions were at least equally prioritized with respect to multidisciplinary treatment at specialized rehabilitation clinics [36].

Second, our findings demonstrated a limited impact of age, being born outside Sweden, pain intensity and mental illness on the relation between education and being selected for rehabilitation programs. These findings indicate that gender bias may be at stake and that the combination of gender and education play a significant role when deciding who is suitable for multidisciplinary rehabilitation.

Third, Sweden is a country well-known for its historical political engagement for achieving increased equality in society. According to Swedish law, the overall goal for health care is that it should be given on equal terms for the whole population [36]. Our findings point in the direction of discrimination against low-educated women in the rehabilitation of chronic pain which is not in accordance with the Swedish law.

More women than men were referred to rehabilitation. An explanation could be simply the fact that the prevalence of pain is higher in women than men [37], [38]. Another explanation could be that men with pain are more often referred to specialist treatment and therefore get more precise diagnoses and treatment than women [7]. However, the patients in our register were assessed at a specialist clinic. Our findings cannot be interpreted as a gender bias against men because we do not know the clinical reasons behind these findings.

In general, our findings are in line with gender-theoretical research emphasizing the importance of taking several power dimensions into account when analyzing possible bias in treatment [39], [40]. Thus, our findings draw attention to the importance of not viewing men and women as static groups but analyzing differences within (and similarities across) the group of men and women. Intersectional approaches mean that dimensions of inequalities do not simply accumulate. Instead one category such as ‘low education’ takes its meaning from another such as ‘gender’ and new hybrids develop as these categories are new hybrid structures which emerge at the intersections of inequality [41]. Qualitative methods could preferably be used in order to understand the meaning of such hybrids in pain rehabilitation. Thus, there is a need for more intersectional research about what happens in the meeting between patients and care-givers. In this study, we have no such measures.

### Limitations and strengths

The current study is based on register data which has some limitations. Above we discuss that our findings point in the direction of discrimination of low-educated women. Discrimination can be seen to exist if high and low educated women have the same health needs but receive different treatment. However, the lack of certain social and clinical variables in the SQRP register about health needs prohibits us from drawing firm conclusions about discrimination. Even though we assume that rehabilitation is the best treatment for the patients referred to the pain rehabilitation clinic, it could be the case that the evaluating teams concluded that the low educated women would not benefit from the programs due to for example manual workload, domestic strain and less possibility to rest. But none of these circumstances are considered contra-indicative of multidisciplinary rehabilitation, and should not be relevant when decisions are taken by multidisciplinary teams. As the rehabilitation programs take into account the individual needs of the patients and support them to set their own goals, we have no reason to believe that the needs of lower educated women are not attended to in the assessment. Since the physicians examine patients by standard procedures we do not believe that mis-diagnosis in women with low education is a problem.

Due to the limitations of the register data we do not have information on diagnoses of diseases causing the pain. However, in a previous study from the pain rehabilitation clinic (with access to diagnoses) no significant differences were found in diagnostic groups between patients being selected for multidisciplinary rehabilitation compared to all assessed patients [16]. In addition, there are strict selection criteria for the pain rehabilitation clinic in our study, which means that in order to get an initial appointment at the clinic, the patients must have a disabling non-malignant diagnosis of chronic pain and that other diagnoses (such as cancer, rheumatoid arthritis, neurological disorders) are excluded.

Co-morbidity could be the basis of different therapeutic efforts in patients with different levels of education. Our register contained information about the most important comorbid conditions, which are depression and anxiety [43], [44]. We have performed sensitivity analyzes with clinical cut-off points for depression and for anxiety (with case level >10 in HADS). The inclusion of the clinical cut-off points for depression and anxiety separately did not change the overall findings. But a limitation is that we do not have information about other comorbidity, for example post-traumatic stress symptoms and fear avoidance. Earlier research shows no socioeconomic differences in post-traumatic stress symptoms [42]. Comorbid symptoms of fear avoidance are not contra-indicative of multidisciplinary pain rehabilitation and are dealt with in rehabilitation programs. A minority of the patients referred to the Pain Rehabilitation Clinic suffered from other physical diseases and were referred to other clinics. As this is a very small group we have no reason to believe that low-educated women are over-represented among them.

Due to these methodological uncertainties, we interpret our findings as a possible (in contrast to a confirmed) discrimination against low-educated women. There is a need for more empirical research about the topic with studies which have more clinical data as well as more information about the decision making process.

More women than men were referred to the rehabilitation clinic. Therefore, lack of significant findings between educational level and selection for multidisciplinary rehabilitation among men may be due to a type 2-error. Use of an already established registry (SQORP) for measures of socio-demographic data and pain indicators restricted the possibility of including other measures of interest. On the other hand, the measures included are validated and have been widely used in clinical practice for assessment of pain severity, anxiety and depression.

Low education was defined as not having education beyond compulsory school, but what is ‘low’ can always be discussed. Sensitivity analyzes were performed with other dichotomizations,such as no education beyond secondary high school, which showed similar results. Therefore, we chose to use the lowest level of educational attainment.

The main strength of the present study is the relatively high number of patients included and that recruitment of participants was restricted to one specific rehabilitation clinic. During the three-year inclusion period, the procedures for multidisciplinary team assessments did not change, thus enhancing the reliability of the data. In addition the team assessment was performed by experienced professionals with high staff continuity during the data collection period. Further, SQRP is a national register for pain rehabilitation and includes approximately 80% of pain management programs in Sweden [17]. The procedure used by the multidisciplinary team for selection of patients for multidisciplinary rehabilitation is similar throughout Sweden; thus, we can assume that the generalisability of the study is good on a national level.

Moreover, since comparable multidisciplinary assessment and selection visits often precede participation in rehabilitation programs in other counties as well [16], and since the MPI and HADS questionnaires have been widely used for measuring chronic pain, depression and anxiety in a range of pain rehabilitation contexts [20], we can assume that the generalisability of the study is good to countries with similar organization for the rehabilitation of patients with chronic pain.

Our outcome measure takes account of both diagnostic (International Classification of Diseases, 10th version (ICD-10)) and functional (International Classification of Disability, Impairment and Handicap (ICIDH)) components [17]. The SQRP consists of validated scales [20], [21]. Selection criteria and assessment procedures for multidisciplinary rehabilitation are relatively similar across countries that offer organized treatment of patients with chronic pain [16]; thus, we can assume that the external validity of the study is relatively good.

In this study general practitioners referred the patients to a specialist pain rehabilitation clinic. Thus, the patients represent a selected group with a more complex chronic pain condition than patients treated in primary care. More research on this topic is needed in other contexts – both other clinical contexts and various geographical locations.

## Conclusions

Our findings can be interpreted as possible discrimination against low-educated women with chronic pain in hospital referrals to multidisciplinary pain rehabilitation. More research is needed to analyze whether such discrimination also occurs in other clinical settings. There is a need for more gender-theoretical research emphasizing the importance of taking several power dimensions into account when analyzing possible bias in treatment.

## References

[1] Risberg G (2004) “I am solely a professional – neutral and genderless”: on gender bias and gender awareness in the medical profession [Dissertation]: Umeå University.

[2] Ruiz-Cantero MT, Vives-Cases C, Artazcoz L, Delgado A, Garcia Calvente MM, et al (2007) A framework to analyse gender bias in epidemiological research. J Epidemiol Community Health 61 Suppl 2ii46–53. 18000118 10.1136/jech.2007.062034 PMC2465769

[3] Bosner S, Haasenritter J, Hani MA, Keller H, Sonnichsen AC, et al (2011) Gender bias revisited: new insights on the differential management of chest pain. BMC Fam Pract 12: 45. 21645336 10.1186/1471-2296-12-45 PMC3125218

[4] Borkhoff CM, Hawker GA, Kreder HJ, Glazier RH, Mahomed NN, et al (2008) The effect of patients' sex on physicians' recommendations for total knee arthroplasty. CMAJ 178: 681–687. 18332383 10.1503/cmaj.071168 PMC2263116

[5] Nyberg F, Osika I, Evengård B (2008) “The Laundry Bag Project” – unequal distribution of dermatological healthcare resources for male and female psoriatic patients in Sweden. Int J Dermatol 47: 144–149. 18211484 10.1111/j.1365-4632.2008.03485.x

[6] Raine R (2000) Does gender bias exist in the use of specialist health care? J Health Serv Res Policy 5: 237–249. 11184961 10.1177/135581960000500409

[7] Hamberg K, Risberg G, Johansson EE, Westman G (2002) Gender bias in physicians' management of neck pain: a study of the answers in a Swedish national examination. J Womens Health Gend Based Med 11: 653–666. 12396897 10.1089/152460902760360595

[8] Noone JH, Stephens C (2008) Men, masculine identities, and health care utilisation. Sociol Health Illn 30: 711–725. 18564976 10.1111/j.1467-9566.2008.01095.x

[9] Apesoa-Varano EC, Hinton L, Barker JC, Unützer J (2010) Clinician Approaches and Strategies for Engaging Older Men in Depression Care. Am J Geriatr Psychiatry 18: 586–595. 20220598 10.1097/JGP.0b013e3181d145ea PMC2981127

[10] Gendered Innovation's homepage. Available: http://genderedinnovations.stanford.edu/. Accessed 14 January 2014.

[11] Jensen IB, Bergström G, Ljungquist T, Bodin L (2005) A 3-year follow-up of a multidisciplinary rehabilitation program for back and neck pain. Pain 115: 273–283. 15911154 10.1016/j.pain.2005.03.005

[12] SBU (2010) Chronic Pain Rehabilitation. Stockholm: Swedish Council of Health Technology Assessment. 28876762

[13] SBU (2006) Methods for treatment of chronic pain. Stockholm: The Swedish Council on Technology Assessment in Health Care. Report No.177.

[14] Scascighini L, Toma V, Dober-Spielmann S, Sprott H (2008) Multidisciplinary treatment for chronic pain: a systematic review of interventions and outcomes. Rheumatology (Oxford) 47: 670–678. 18375406 10.1093/rheumatology/ken021

[15] Löfmark U, Hammarström A (2007) Evidence for age-dependent education-related differences in men and women with first-ever stroke. Results from a community-based incidence study in northern Sweden. Neuroepidemiology 28: 135–141. 17478968 10.1159/000102141

[16] Merrick D, Sundelin G, Stålnacke BM (2012) One-year follow-up of two different rehabilitation strategies for patients with chronic pain. J Rehabil Med 44: 764–773. 22915044 10.2340/16501977-1022

[17] Nyberg V, Sanne H, Sjölund BH (2011) Swedish quality registry for pain rehabilitation: purpose, design, implementation and characteristics of referred patients. J Rehabil Med 43: 50–57. 21042698 10.2340/16501977-0631

[18] Zigmond AS, Snaith RP (1983) The hospital anxiety and depression scale. Acta Psychiatr Scand 67: 361–370. 6880820 10.1111/j.1600-0447.1983.tb09716.x

[19] Cosco TD, Doyle F, Ward M, McGee H (2012) Latent structure of the Hospital Anxiety and Depression Scale: a 10-year systematic review. J Psychosom Res 72: 180–184. 22325696 10.1016/j.jpsychores.2011.06.008

[20] Bjelland I, Dahl AA, Haug TT, Neckelmann D (2002) The validity of the Hospital Anxiety and Depression Scale. An updated literature review. J Psychosom Res 52: 69–77. 11832252 10.1016/s0022-3999(01)00296-3

[21] Kerns RD, Turk DC, Rudy TE (1985) The West Haven-Yale Multidimensional Pain Inventory (WHYMPI). Pain 23: 345–356. 4088697 10.1016/0304-3959(85)90004-1

[22] Pietila Holmer E, Fahlström M, Nordström A (2013) The effects of interdisciplinary team assessment and a rehabilitation program for patients with chronic pain. Am J Phys Med Rehabil 92: 77–83. 23255272 10.1097/PHM.0b013e318278b28e

[23] Dworkin RH, Turk DC, Farrar JT, Haythornthwaite JA, Jensen MP, et al (2005) Core outcome measures for chronic pain clinical trials: IMMPACT recommendations. Pain 113: 9–19. 15621359 10.1016/j.pain.2004.09.012

[24] Beswick AD, Rees K, West RR, Taylor FC, Burke M, et al (2005) Improving uptake and adherence in cardiac rehabilitation: literature review. J Adv Nurs 49: 538–555. 15713186 10.1111/j.1365-2648.2004.03327.x

[25] Carr JL, Moffett JA (2005) The impact of social deprivation on chronic back pain outcomes. Chronic Illn 1: 121–129. 17136918 10.1177/17423953050010020901

[26] Melville MR, Packham C, Brown N, Weston C, Gray D (1999) Cardiac rehabilitation: socially deprived patients are less likely to attend but patients ineligible for thrombolysis are less likely to be invited. Heart 82: 373–377. 10455092 10.1136/hrt.82.3.373 PMC1729163

[27] van den Bos GA, Smits JP, Westert GP, van Straten A (2002) Socioeconomic variations in the course of stroke: unequal health outcomes, equal care? J Epidemiol Community Health 56: 943–948. 12461116 10.1136/jech.56.12.943 PMC1756981

[28] van Doorslaer E, Masseria C, Koolman X (2006) Inequalities in access to medical care by income in developed countries. CMAJ 174: 177–183. 16415462 10.1503/cmaj.050584 PMC1329455

[29] Kapral MK, Wang H, Mamdani M, Tu JV (2002) Effect of socioeconomic status on treatment and mortality after stroke. Stroke 33: 268–273. 11779921 10.1161/hs0102.101169

[30] Björnsdottir SV, Jonsson SH, Valdimarsdottir UA (2013) Functional limitations and physical symptoms of individuals with chronic pain. Scand J Rheumatol 42: 59–70. 23126682 10.3109/03009742.2012.697916

[31] Friedrich M, Hahne J, Wepner F (2009) A controlled examination of medical and psychosocial factors associated with low back pain in combination with widespread musculoskeletal pain. Phys Ther 89: 786–803. 19541773 10.2522/ptj.20080100

[32] Macfarlane GJ, Norrie G, Atherton K, Power C, Jones GT (2009) The influence of socioeconomic status on the reporting of regional and widespread musculoskeletal pain: results from the 1958 British Birth Cohort Study. Ann Rheum Dis 68: 1591–1595. 18952642 10.1136/ard.2008.093088

[33] Perruccio AV, Gandhi R, Rampersaud YR (2013) Heterogeneity in health status and the influence of patient characteristics across patients seeking musculoskeletal orthopaedic care – a cross-sectional study. BMC Musculoskelet Disord 14: 83. 23497192 10.1186/1471-2474-14-83 PMC3599249

[34] Toivanen S (2011) Exploring the interplay between work stress and socioeconomic position in relation to common health complaints: the role of interaction. Am J Ind Med 54: 780–790. 21692098 10.1002/ajim.20982

[35] Overland S, Harvey SB, Knudsen AK, Mykletun A, Hotopf M (2012) Widespread pain and medically certified disability pension in the Hordaland Health Study. Eur J Pain 16: 611–620. 22396089 10.1016/j.ejpain.2011.08.005

[36] Health and Medical Services Act [Hälso- och sjukvårdslagen] SFS (1982) 763.

[37] Winjhoven HA, deVet HC, Picavet HC (2006) Prevalence of musculoskeletal disorders is systematically higher in women than in men. Clin J Pain 22: 717–24. 16988568 10.1097/01.ajp.0000210912.95664.53

[38] Stubbs D, Krebs E, Bair M, Damush T, Wu J, et al (2010) Sex-differences in pain and pain related disability among primary care patients with chronic musculoskeletal pain. Pain Med 11: 232–9. 20002591 10.1111/j.1526-4637.2009.00760.x

[39] Hankivsky O (2012) Women's health, men's health, and gender and health: implications of intersectionality. Soc Sci Med 74: 1712–1720. 22361090 10.1016/j.socscimed.2011.11.029

[40] Springer KW, Hankivsky O, Bates LM (2012) Gender and health: relational, intersectional, and biosocial approaches. Soc Sci Med 74: 1661–1666. 22497844 10.1016/j.socscimed.2012.03.001

[41] Shields S (2008) Gender: An intersectionality perspective. Sex Roles 59: 301–311.

[42] Boman KK, Kjällander Y, Eksborg S, Becker J (2013) Impact of prior traumatic life events on parental early stage reactions following a child's cancer. PLoS One 8(3): e57556. 23516408 10.1371/journal.pone.0057556 PMC3597714

[43] Bair MJ, Robinson RL, Katon W, Kroenke K (2003) Depression and pain comorbidity: a literature review. Arch Intern Med 163: 2433–2445. 14609780 10.1001/archinte.163.20.2433

[44] Jordan KD, Okifuji A (2011) Anxiety disorders: differential diagnosis and their relationship to chronic pain. J Pain Palliat Care Pharmacother 25: 231–245. 21882977 10.3109/15360288.2011.596922

